# Predictors of Stunting among School-Age Children in Northwestern Ethiopia

**DOI:** 10.1155/2018/7521751

**Published:** 2018-09-20

**Authors:** Amare Lisanu Mazengia, Gashaw Andargie Biks

**Affiliations:** ^1^College of Health Sciences, Salale University, Fiche, Ethiopia; ^2^College of Health Sciences and Medicine, Gondar University, Gondar, Ethiopia

## Abstract

**Background:**

Stunting is a major public health problem in most developing countries, and it increases the risk of illness and death throughout childhood. It is also a major public health problem in Ethiopia. Most of the few studies done in Ethiopia were done in schools. However, the prevalence of stunting of school-age children at the community level is largely unknown.

**Objective:**

To assess prevalence and predictors of stunting among school-age children in Mecha District, Amhara Regional State, Ethiopia.

**Methods:**

A community-based cross-sectional study was conducted from August 28, 2017, to October 10, 2017. Target population for the study was school-age children (children of age 5–15). A total of 802 children were included in the study. The multistage sampling procedure was applied. Data were collected through face-to-face interview using the structured questionnaire. Anthropometric measurement was performed and analyzed using ENA SMART software. Association was assessed using logistic regression (backward LR). Statistical significance was measured using adjusted odds ratio at 95% CI and *P* value less than 0.05.

**Results:**

About 37.9%, with 95% CI (34.6, 41.3), of children were stunted. The predictors of stunting were child age with AOR (95% CI) 1.4 (1.02, 1.91), family size with AOR (95% CI) 1.83 (1.21, 2.75), mother's education with AOR (95% CI) 1.81 (1.01, 3.24), father's occupation with AOR (95% CI) 5.23 (1.55, 17.64), and child's immunization status with AOR (95% CI) 5.6 (2.90, 10.82).

**Conclusion:**

Stunting is still an important problem among children in the age of 5–15 years. Therefore, special attention should be given to child's full immunization, limiting of family size, continued promotion of female education, and appropriate feeding practice of children depending on their age.

## 1. Background

Malnutrition continues to be a major public health problem in developing countries [1]. It is the most important risk factor for the burden of disease directly and indirectly responsible for more than half of all deaths in children [1–3]. About 53% of child deaths are attributable to malnutrition in the world [4]. Stunting is one form of malnutrition, and children whose height-for-age *Z*-score is below −2 SD from the median of WHO reference population are considered short for their age (stunted). Children who are below −3 SD are considered severely stunted [5, 6].

Stunting reveals miscarriage to obtain sufficient amount and quantity of nutrition over a long period of time and is influenced by environmental factors, socioeconomic circumstances, and recurrent and chronic illness [6]. Therefore, stunting represents the long-standing effects of nutritional problems in the community and is not sensitive to recent, short-term deviations of dietary intake [7]. Stunting leads to 14.5% of deaths yearly and 12.6% of infirmity adjusted life-years [8]. Stunting decreases future school achievements and revenue as adults, and it raises the threat of obesity and noncommunicable diseases [9].

Globally, the estimated number of stunted under-five children is 165 million [10]. More than 200 million school-age children were stunted by the year 2000, and the proportion of stunted school-age children with impaired physical and mental development is expected to grow up to 1 billion by the year 2020 unless a tangible action is undertaken [11].

About 25% of African elementary schoolchildren were found below the fifth centile of the United States-National Center for Health Statistics (US-NCHS) reference for height-for-age *Z*-score (HAZ) [12]. In Ethiopia, 40 percent of under-five children are stunted, and 18 percent of children are severely stunted. In the Amhara region, 42 percent of under-five children are stunted [13]. Even though stunting has serious consequences on morbidity and mortality of children, limited attention has been given [14], and cautious attention of stunting indicator is an important part of policy discussions on community nutrition programs [15]. Stunting is not considered in school-age children in many parts of the world including Ethiopia, and little is known about the occurrence of stunting in this age group. Therefore, to develop effective interventions and to decrease stunting in this age group of children, a sound understanding on the magnitude and predictors of stunting is essential.

## 2. Methods

### 2.1. Study Design Area and Period

A quantitative community-based cross-sectional study was conducted to assess prevalence and predictors of stunting among school-age children. This study was conducted at the Mecha district from August 28, 2017, to October 10, 2017. The Mecha district is located 529 km northwest away from the capital, Addis Ababa, and it is situated at an altitude ranging from 1800 m to 2500 m. The district receives an average annual rainfall ranging from 1000 mm to 2000 mm and average daily temperature from 17°C to 30°C. The total population of the district was about 370032; of these, 80727 (21.8%) were in the age of 5–15 years [16].

### 2.2. Source Population and Sample Size Determination

The source population was all children from the age of 5–15 years who live in the Mecha district, and the study population was all children from the age of 5–15 years who live in the selected kebeles. Sample size was determined by using a single population proportion formula with the assumptions of 95% CI, 5% of margin of error, reported prevalence of stunting in school-age children *P* (48.1%) [17], design effect 2, and 5% possible nonresponse rate, which gives the total sample size of 802.

### 2.3. Inclusion and Exclusion Criteria

All children aged between 5 and 15 years who live in the district were included in the study. Children who had anatomical deformity, seriously ill children during data collection, and children whose age was not known were excluded.

### 2.4. Sampling Technique

The multistage simple random sampling procedure was used with the strata of kebeles which exist in semiurban and rural. A total of 46 kebeles are in the district (40 rural andand 6 semiurban). At the first stage, due to resource constraints, 20% of the kebeles (8 rural and 1 semiurban kebeles proportionally) were selected by using the simple random sampling technique. Then, the total sample was allocated proportionally to the study population size of each kebele. By using family folder lists (CHIS Household ID) of the health extension program, the sampling frame was prepared, the simple random sampling technique was applied, and then participants from each kebele were identified by using the computer randomized method.

### 2.5. Variables of the Study

#### 2.5.1. Dependent Variable

Children with *Z*-score less than −2 (height for age < −2) were categorized as stunted, whereas those with *Z*-score −2 and above were categorized as normal as per the 2007 WHO *Z*-score standard.

#### 2.5.2. Independent Variables

Sociodemographic variables: marital status, educational status, occupation, place of residence (being rural or semiurban), family size, and household assets. Child characteristics: age, sex, height, gestational age during birth, exclusive breastfeeding status, and morbidity status. Child-caring practices: hygiene, feeding, health care seeking, and immunization. Maternal characteristics: total number of children and sovereignty in decision-making on use of resources. Environmental health condition: availability of latrine, water supply, sanitation, and housing condition.

### 2.6. Data Collection and Quality Control

Data were collected using a pretested structured questionnaire by face-to-face interview. The interviews were conducted by six freshly graduated trained clinical nurses supervised by two health officers. Standard UNICEF-aided fixed board meters were used to measure the height of the children. Measurement of height was taken to the nearest 0.1 cm with the children barefooted. Data collectors and supervisors were trained on the use of the fixed board meters prior to the fieldwork.

The age of children was collected in months, and if the mother could not remember child's age, local events were used to assist the mother. Supervisors and principal investigator (PI) checked the accuracy and completeness of the data that were collected daily. Any error in data collection was corrected before proceeding with the next day's data collection activity. The interview was conducted in a place where the participants felt free to express themselves. To assure the data quality, one-day training was given to the data collectors as well as the supervisors concerning the objectives of the study, ways of the interview, the questionnaire, and how to measure each child's height with demonstration. The data collection tool was pretested prior to the real data collection on 40 participants (5% of the sample), in a place other than the study area. This was a good chance to make changes in the questionnaire which was difficult to understand during the interview. Throughout the progression of data collection, data collectors were overseen at each site of data collection place, and daily meetings were conducted between the data collectors and the principal investigator in such a way that problematic issues faced during the interview were discussed and decisions were reached. One additional visit was done for respondents who were not found in the first visit. The gathered data were revised and verified for completeness prior to data entry; the incomplete data were excluded.

### 2.7. Data Processing and Analysis

The complete data were coded and entered into Epi Info (version 3.5.1), and they were exported to SPSS version 20 for analysis. Anthropometric data were analyzed using ENA SMART software in terms of the WHO standard, and the wealth index was analyzed using Stata (version 12). Data cleaning was made manually to exclude subjects with incomplete data. Reliability of the cleaned data was checked through Cronbach's alpha, and its value is 0.71 which is greater than the cutoff point. The final data file was compiled for analysis. During the analysis, frequencies of different variables were determined, followed by cross-tabulation to compare the frequencies. Binary logistic regression analysis was done to measure the crude association between each independent variable through dependent variable, and variables having *P* value <0.2 were included in multivariable logistic regression analysis. The Hosmer and Lemeshow test for model fitness was checked in multivariable logistic regression, and it is 0.94 which is much greater than the cutoff point. *P* value <0.05 was used as a cutoff point for statistical significance of the study.

### 2.8. Ethical Approval

The proposal was reviewed and approved by the Research and Ethics Committee of the University of Gondar. The district health office was informed by an official letter from the University of Gondar, and permission to undertake this study was obtained from the concerned authorities. Then, participants were informed about the importance of the study, necessity of their contribution, risk, and discontinuation at any time, and written consent and assent were obtained before the data collection. Confidentiality of data given by each participant was maintained. After completing the interview, acknowledgment was given to the participants, and seriously ill individuals were referred to the nearest health institutions.

### 2.9. Operational Definitions

  Stunting: children who have height for age below −2 SD of the median of the standard curve (WHO reference population).
  School-age children: children in the age of 5–15 years.
  Timely started complementary food: diets which are essential to the child and started at six months of age.
  Wealth index (WI): It is a merged index collected of important asset possession variables used as a representation indicator of the family level of wealth [18].

## 3. Results

### 3.1. Sociodemographic Characteristics

From all 802 participants included in this study, 800 (99.75%) delivered complete response. Altogether, 728 (91%) of households were headed by males who were married. Majority (733: 91.6%) of respondents lived in rural areas. Out of the respondents, 597 (74.6%) of households had more than national average family size which is greater than or equal to five. Concerning educational status, 664 (83%) of mothers and 597 (74.6%) of fathers were illiterate (could not read and write). Around 690 (86.3%) of mothers and 677 (84.7%) of fathers were farmers. About 544 (68%) of both husband and wife decided equally on use of household resources. Concerning livestock, 713 (89.1%) of families had livestock and 691 (86.4%) of households had farm land, and from these, 584 (73%) had >0.5 hectare (Table 1).

### 3.2. Children Characteristics and Caring Practices

Among the total children, 458 (57.2%) were in the age of 60–120 months and 342 (42.8%) were in the age of 121–180 months, and 419 (52.4%) of the total participants were males. The mean age and SD of school-age children in the current study were 115.3 and 32.5, respectively. About 19 (2.4%) of children were born after less than nine months of gestation. Regarding complementary feeding practices, only 394 (49.3%) were started appropriately at six months. The most frequent child-feeding practice was three times per day (471 (58.9%); Table 2).

### 3.3. Environmental Health Characteristics of Households

Regarding treatment of water, about 771(96.4%) of HHs did not treat water by any means for their family consumption. Most family units (711 (88.9%)) had la- 6trines, and 499 (62.4%) of families used only water for handwashing after toilet. About the waste disposal system, 476(59.5%) households disposed their garbage in open field,while 236 (29.5%) used individual pits and/or composts. Themain sources of drinking water in the households wereunprotected sources (474 (59.3%); spring, well, and riverwater), and 326 (40.7%) were from protected spring/well andpublic tab (Table 3)

### 3.4. Stunting/Height-for-Age

From 60 to 180 months, participants (303 (37.9%); 95% CI (34.6, 41.3)) were stunted. No significant difference in stunting was identified between boys (159 (19.9%)) and girls (144 (18%)) in this study. Children from educated mothers were less likely to be stunted (22 (2.8%)) than those from uneducated mothers (281 (35.1%)). Severe stunting was observed in 84 (10.5%; 95% CI (8.6, 12.8)) children: the frequency was almost equal in males (41 (5.1%)) and females (43 (5.4%)).

### 3.5. Factors Associated with Stunting of Children

As presented in Tables 4 and 5, first predictors were assessed through binary logistic regression analysis: of these, twelve variables had significant effects on stunting. Variables that had *P* value <0.2 in binary logistic regression were accepted into multivariable logistic regression analysis. Among these, five predictors were statistically significant (*P* < 0.05).

## 4. Discussion

The overall prevalence of stunting in the current study was about 38%. This prevalence rate was slightly lower than 40% which is often cited as the cutoff point for public health significance. It was higher than figures reported in facility-based cross-sectional studies conducted worldwide (26%) [11], eight provinces of Indonesia (28.11%) [19], Palestine (7.3%) [20], Baghdad, Iraq (18.7%) [21], Rongshui County and Dingan County of Southern China (25.6%) [22], Onda, Bankura District, India (17%) [23], and Sagamu in Southwestern Nigeria (14.2%) [24]. The observed variance in rates might be due to differences in study area, socioeconomical, topographical, developmental, cultural, and feeding habits of children.

The prevalence of stunting in the current study was also much higher than that in other studies that were reported in Ethiopia—11.5% in Horo Guduru Welega, 25.2% in Dale Woreda, Sidama Zone, and 25.2% in two randomly selected governmental elementary schools in Gondar town. This difference may have arisen partly from peculiarities of the target populations in the various studies considering that the current study was community-based, while the earlier ones were among schoolchildren. It is conceivable that stunted children were more likely to drop out of school and would remain in the community.

On the contrary, some earlier Ethiopian studies reported rates of stunting comparable to or higher than those found in the current study—39.8% and 48.1% from Libo Kemkem and Fogera Woreda [25] and East Gojjam Zone [17], respectively. Also, a study from Indonesia [19] reported a higher rate of 46.5%. Explanations for the differences in rates could lie in cultural, socioeconomical, and geographical factors.

The current study found that the odds of stunting were 1.4 times higher in children above 10 years than in younger subjects. This pattern is similar to earlier findings in Indonesia [19] and other parts of Ethiopia like Dale Woreda [26] and Fogera Woreda and Libo Kemkem [25]. This reflects the nature of stunting as a chronic nutritional problem which develops over a relatively long period of time and which is difficult to reverse once established.

A strong relationship was established in the present study between lack of immunization and stunting. The reasons for this association were not investigated and are more likely to be indirect than direct. It may be argued that poorly vaccinated children are more likely to contract illnesses that may in turn lead to stunting. It is also plausible that the same socioeconomic and cultural factors responsible for stunting may also influence the attitude and practice of parents with respect to child immunization.

In line with findings in Indonesia [19], large family size was found to be strongly associated with stunting. The large family size would imply lower quantity of food available for each family member in comparison with smaller families with the same economic power. Moreover, large family size may also increase the risk of overcrowding that will lead to spreading of diseases such as recurrent acute respiratory infections, malaria, and diarrhea which can lead to malnutrition. Therefore, it is not surprising that stunting occurred more commonly among children from large number families compared to children from small number families.

In this study, illiterate mother was one of the risk factors of stunting. Maternal education has been previously found to be an important risk factor for stunting in Indonesia [19], in Southern China [22], and in Abeokuta, Southwest Nigeria [27]. Educated mother may be more exposed to the media, not illiterate, have higher participation in the labor market, and have better understanding on nutrition and health. She may also have greater authority in the home and can raise productivity to improve their family and child nutritional status. She would also better use the childhood survival strategies, like sufficient breastfeeding, vaccination, and limiting of their family size. As a result, education of girls would be the wise method of reducing the magnitude of stunting.

Paternal occupation was also another significantly associated factor of stunting. This had been reported in an earlier Fincha's sugar estate, an Ethiopian study as well [28]. Moreover, the results of the current study indicate no association between wealth index and stunting. Although the wealth index is taken as a proxy for household assets, the apparently contradictory finding may have resulted from withholding of relevant information on assets and/or father's education.

## 5. Conclusion

The magnitude of stunting in the study area was very high but fell slightly short of the cutoff point for being classified as a public health concern. Occupational status of fathers, educational status of mothers, family size, immunization level, and age of children were significant predictors of stunting in school-age children. These findings suggest the need for strengthening childhood immunization, limiting of family size, improved female education, and appropriate feeding practice of children.

## Figures and Tables

**Table 1:** Sociodemographic characteristics of participants at Mecha district, Northwestern Ethiopia, 2017 (*N* = 800).

Variables	Category	Frequency	Percentage
Residence	Rural	733	91.6
	Semiurban	67	8.4
Family size	≥5	597	74.6
	<5	203	25.4
Mother's educational level	Illiterate	664	83
	Literate	136	17
Father's educational level	Illiterate	597	74.6
	Literate	202	25.4
Decision-maker on use of money	Mainly wife	74	9.3
	Mainly husband	182	22.7
	Both jointly	544	68
Marital status of mother	Married	728	91
	Divorced	47	5.9
	Others^*∗*^	25	3.1
Mother's occupation	Farmer/housewife	690	86.3
	Merchant	96	12
	Governmen't and private employee	14	1.8
Father's occupation	Farmer	677	84.6
	Merchant	75	9.4
	Government and private employee	48	6

^*∗*^Widowed and single.

**Table 2:** Characteristics of school-age children and their mothers' practice to give childcare at Mecha district, Northwestern Ethiopia, 2017 (*N* = 800).

Variable	Category	Frequency	Percentage
Sex of child	Male	419	52.4
	Female	381	47.6
Child's age in months	60–120	458	57.2
	121–180	342	42.8
Gestational age of child during birth	<9 months	19	2.4
	≥9 months	781	97.6
Child's starting age of complementary feeding	<6 months	120	15
	>6 months	286	35.8
	At 6 months	394	49.2
Fully immunized child	No	59	7.4
	Yes	741	92.6
Age of the mother at first birth	<19 years	16	2.5
	≥19 years	612	97.5
Total number of children in the family	<4	314	39.3
	≥4	486	60.7
Family planning used	No	246	30.7
	Yes	554	69.3
Types of family planning used	Short term	510	63.8
	Long term and permanent	44	5.5

**Table 3:** Environmental health characteristics of households at Mecha district, Northwestern Ethiopia, 2017 (*N* = 800).

Variable	Category	Frequency	Percentage
Do you treat drinking water by any means	No	771	96.4
	Yes	29	3.6
Have you a latrine	No	89	11.1
	Yes	711	88.9
Hand washing practice of child's mother after toilet	Not washed	53	6.6
	Using water only	499	62.4
	Sometimes using soap/ash	155	19.4
	Always using soap/ash	93	11.6
Methods of HHs' waste disposal	Open-field disposal	476	59.5
	Individual pit/compost	236	29.5
	Common pit	28	3.5
	Burning	60	7.5

**Table 4:** Binary logistic regression analysis results in school-age children at Mecha district, Northwestern Ethiopia, 2017 (*N* = 800).

Variables	Stunted (*N* = 303)	Nonstunted (*N* = 497)	COR (95% CI)	*P* values
Residence				
Rural	295	438	4.97 (2.34, 10.55)	*P* < 0.0001
Semiurban	8	59	1.00	

Age of children				
121–180 months	149	193	1.52 (1.14, 2.03)	*P* = 0.004
60–120 months	154	304	1.00	

Fully immunized child				
No	46	13	6.66 (3.54, 12.6)	*P* < 0.0001
Yes	257	484	1.00	

Source of drinking water				
Unprotected source	194	280	1.4 (1.03, 1.85)	*P* = 0.032
Protected source	109	217	1.00	

Hand washing practice of child's mother after toilet				
Not washed	25	28	2.43 (1.20, 4.93)	*P* = 0.014
Using only water	190	309	1.67 (1.02, 2.74)	*P* = 0.041
Sometimes soap/ash	63	92	1.86 (1.07, 3.26)	*P* = 0.029
Always soap/ash	25	68	1.00	

Wealth index				
Quintile 1	56	104	2.15 (1.30, 3.57)	*P* = 0.003
Quintile 2	74	86	3.44 (2.10, 5.66)	*P* < 0.0001
Quintile 3	70	90	3.11 (1.89, 5.12)	*P* < 0.0001
Quintile 4	71	89	3.19 (1.94, 5.25)	*P* < 0.0001
Quintile 5	32	128	1.00	

Family size				
≥5	249	338	2.17 (1.53, 3.08)	*P* < 0.0001
<5	54	159	1.00	

Father's education				
Illiterate	242	355	1.59 (1.13, 2.23)	*P* = 0.008
Literate	61	142	1.00	

Mother's education				
Illiterate	281	383	3.80 (2.35, 6.15)	*P* < 0.0001
Literate	22	114		

Mother's occupation				
Housewife/farmer	283	407	9.04 (1.18, 69.49)	*P* = 0.034
Merchant	19	77	3.21 (0.39, 26.07)#	*P* = 0.276
Govern't and private worker	1	13	1.00	

Father's occupation				
Farmer	290	387	8.24 (2.93, 23.20)	*P* < 0.0001
Merchant	9	66	1.50 (0.44, 5.17)^#^	*P* = 0.521
Government and private worker	4	44	1.00	

Types of FP used				
Not used modern FP	104	142	3.30 (1.47, 7.39)	*P* = 0.004
Short term	191	319	2.69 (1.23, 5.92)	*P* = 0.014
Long term andand permanent	8	36	1.00	

*Note.* ^#^Not significant in binary logistic regression; COR: crude odds ratio.

**Table 5:** Predictor variables of multivariable logistic regression analysis in school-age children at Mecha district, Northwestern Ethiopia, 2017 (*N* = 800).

Variables	Stunted (*N* = 303)	Nonstunted (*N* = 497)	COR	AOR (95% CI); *P* values
Age of children				
121–180 months	149	193	1.52	1.4 (1.02, 1.91); *P* = 0.037
60–120 months	154	304	1.00	

Fully immunized child				
No	46	13	6.66	5.6 (2.90, 10.82); *P* < 0.0001
Yes	257	484	1.00	

Family size				
≥5	249	338	2.17	1.83 (1.21, 2.75); *P* = 0.004
<5	54	159	1.00	

Mother's education				
Illiterate	281	383	3.80	1.81 (1.01, 3.24); *P* = 0.045
Literate	22	114		

Father's occupation				
Farmer	290	387	8.24	5.23 (1.55, 17.64); *P* = 0.008
Merchant	9	66	1.50	1.49 (0.42, 5.3); *P* = 0.5^*∗∗*^
Government and private worker	4	44	1.00	

*Note.* ^*∗∗*^Not significant; COR: crude odds ratio; AOR: adjusted odds ratio.

## Data Availability

The data supporting the findings of this study are available from the corresponding author upon request.

## Abbreviations

EDHS: Ethiopia Demographic and Health Survey
HAZ: Height-for-age *Z*-score
HHs: Households
SRS: Simple random sampling
WHO: World Health Organization.

## Additional Points

*Limitations of the Study*. Participants' might have a memory loss during responding questions that were asked for the past events. Information on some significant variables might not have been collected, and this could have affected interpretation of results. One example is parasitic infestation which is widespread among children. About 92% of study participants were from rural communities: this limits generalization to urban dwellers. *Recommendations*. Community: mandatory childhood immunization. Family: limiting of family size to an affordable number, and age-specific attention should be given while childfeeding. Administrators: improved access to childhood immunization and sustainable encouragement of female education. Researchers: further studies should be done exploring other potential associated factors that were not included in the present study especially parasitic infection, anemia, and so on.

## References

[1] Müller O., Krawinkel M. (2005). Malnutrition and health in developing countries. *Canadian Medical Association Journal*.

[2] Amy L, Rice S, Hyder A., Blac R. (2000). Malnutrition as an underlying cause of childhood deaths associated with infectious diseases in developing countries. *Bulletin of the World Health Organization*.

[3] Pelletier D. L., Frongillo E. A., Schroeder D. G., Habicht J. P. (1995). The effects of malnutrition on child mortality in developing countries. *WHO Bulletin OMS*.

[4] Caulfield L., Onis M., Blössner M., Black R. (2004). Undernutrition as an underlying cause of child deaths associated with diarrhea, pneumonia, malaria, and measles1. *American Journal of Clinical Nutrition*.

[5] WHO (1995). *Physical Stat Use, the Use and Interpretation of Anthropometry*.

[6] Central Statistical Agency Addis Ababa E (2011). *Ethiopia Demographic and Health Survey 2011*.

[7] WHO (1996). *Catalogue of Health Indicators; A Selection of Important Health Indicators Recommended by WHO Program*.

[8] Victora C., Adair L., Fall C. (2008). Maternal and child undernutrition: consequences for adult health and human capital. *The Lancet Series*.

[9] Pelletier D., Frongillo E. (2003). Community and international nutrition, changes in child survival are strongly associated with changes in malnutrition in developing countries. *Journal of Nutrition*.

[10] Neufeld L. M., Osendarp S. J. (2014). Global, regional and country trends in underweight and stunting as indicators of nutrition and health of populations. *Nestlé Nutrition Institute Workshop Series*.

[11] Dangour A., Uauy R. (2006). Nutrition challenges for the twenty-first century. *British Journal of Nutrition*.

[12] Stefano G. F., De Angelis F. (2009). Anthropometric growth pattern in Ethiopian infants and children: an evaluation based on different international growth references. *Collegium Antropologicum*.

[13] Ethiopia CSAO (2014). *Ethiopia Mini Demographic and Health Survey 2014*.

[14] WHO (2005). *Nutrition for Health and Development, Protection of the Human Environment*.

[15] Lewit E., Kerrebrock N. (1997). Population-based growth stunting. *Future of Children*.

[16] MDH O, Mecha District Health Office Profile, in Office WH, editor, 2014/2015

[17] Zelellw D., Gebreigziabher B., Alene K., Negatie B., Kasahune T. (2014). Prevalence and associated factors of stunting among schoolchildren, in Debre Markos Town and Gozamen Woreda, Ethiopia, 2013. *Journal of Nutrition & Food Sciences*.

[18] Rutstein Shea K. J. (2004). The DHS wealth index.

[19] Yasmin G., Kustiyah L., Dwiriani C. (2014). Risk factors of stunting among school-aged children from eight provinces in Indonesia. *Pakistan Journal of Nutrition*.

[20] Mikki N., Abdul-Rahim H., Awartani F., Holmboe-Ottesen G. (2009). Prevalence and sociodemographic correlates of stunting, underweight, and overweight among Palestinian school adolescents (13–15 years) in two major governorates in the West Bank. *BMC Public Health*.

[21] Al-Saffar A. (2009). Stunting among primary-school children: a sample from Baghdad, Iraq. *Eastern Mediterranean Health Journal*.

[22] Shang Y., Tang L., Zhou S. (2010). Stunting and soil-transmitted-helminth infections among school-age pupils in rural areas of southern China. *Parasites & Vectors*.

[23] Bose K., Bisai S., Mukherjee S. (2013). Anthropometric characteristics and nutritional status of rural school children. *Internet Scientific Publications*.

[24] Fetuga M., Ogunlesi T., Adekanmb A., Alab A. (2011). Nutritional status of semi-urban Nigerian school children using the 2007 WHO reference population. *West African Journal of Medicine*.

[25] Herrador Z., Sordo L., Gadisa E. (2014). Malnutrition and associated factors among school aged children in rural and urban settings of Fogera and Libo Kemkem Districts, Ethiopia. *PLoS One*.

[26] Wolde M., Berhan Y., Chala A. (2015). Determinants of underweight, stunting and wasting among schoolchildren. *BMC Public Health*.

[27] Senbanjo I. O., Oshikoya K. A., Odusanya O. O., Njokanma O. F. (2011). Prevalence of and risk factors for stunting among school children and adolescents in Abeokuta, southwest Nigeria. *Journal of Health, Population and Nutrition*.

[28] Mekonnen Z., Meka S., Zeynudin A., Suleman S. (2014). Schistosoma mansoni infection and under nutrition among school age children in Fincha’a sugar estate, rural part of West Ethiopia. *BMC Research Notes*.

